# Shewanella algae, an Emerging Human Pathogen: A Series of Four Cases From a Portuguese Hospital

**DOI:** 10.7759/cureus.33686

**Published:** 2023-01-12

**Authors:** Salomão Fernandes, Rita Sérvio, Ana Rita Silva, Raquel Tavares, Paulo Rodrigues

**Affiliations:** 1 Infectious Diseases, Hospital Beatriz Ângelo, Loures, PRT

**Keywords:** hiv, antibiotics, migrants, shewanella infection, shewanella algae

## Abstract

*Shewanella algae *is a rod-shaped, Gram-negative bacterium that is considered an emerging human pathogen. Traditionally associated with warmer climates, *S. algae* has now been isolated from patients worldwide, and reports of infection are increasing. In a regional hospital on the outskirts of Lisbon, Portugal, four cases have been detected in the past 10 years. Two of the patients were migrants from African countries with daily contact with water; the other two patients were Portuguese, and no epidemiological risk factors were found among them. These are the first cases reported in Portugal. Risk factors associated with *S. algae infection* in patients discussed in this paper include the following: human immunodeficiency virus (HIV) infection, chronic venous insufficiency, lower limb ulcers, chronic kidney disease, diabetes, arterial hypertension, dilated cardiomyopathy, atrial fibrillation, chronic hepatic disease, and chronic pancreatitis. One patient died in the intensive care unit with septic shock and disseminated intravascular coagulation from a fulminant infection secondary to *S. algae *bacteraemia. The four clinical cases presented in this case series highlight the clinical features of this infection so that other physicians can successfully identify and treat *S. algae *infections.

## Introduction

*Shewanella algae* is a rod-shaped, Gram-negative, aerobic, and motile bacterium from the genus Shewanella. It is a ubiquitous microorganism usually found in seawater, freshwater, and soil, capable of survival under suboptimal environmental conditions [1-3]. Although more than 70 species of Shewanella have been identified, only *S. algae*, *S. putrefaciens*, and *S. xiamenensis* have been associated with human disease, with *S. algae* comprising most cases [4-6].

Traditionally associated with warmer climates, *S. algae* has now been isolated from patients worldwide (including Europe and North America) [1,7], and is considered an emerging human pathogen [6-8]. In this paper, we present a series of four patients infected with *S. algae*, detected over the past 10 years at a regional hospital on the outskirts of Lisbon.

## Case presentation

Case 1

**Figure 1:**
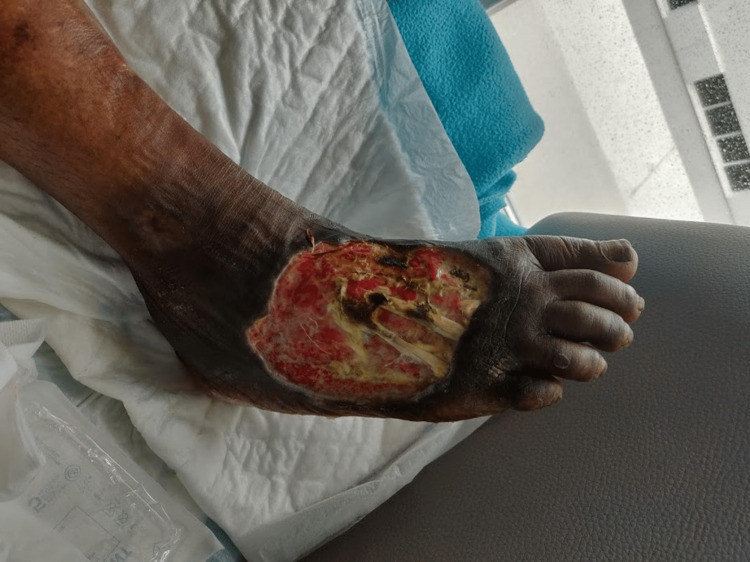
Right foot ulcer upon admission to the emergency department. Pus from the ulcer was collected and Shewanella algae isolated.

**Figure 2:**
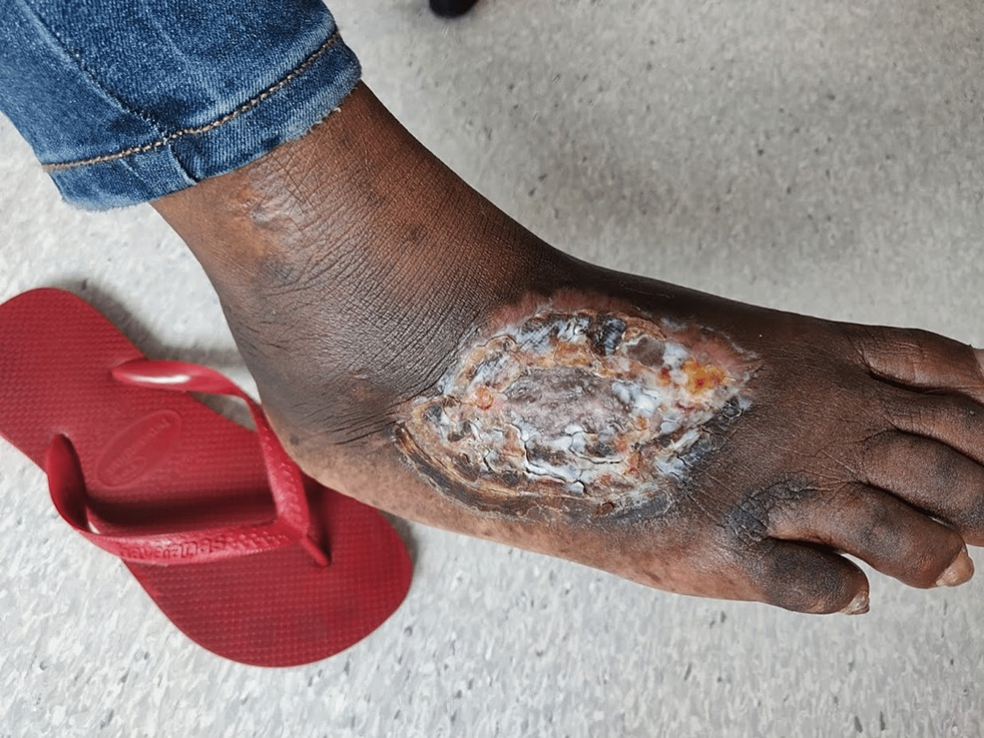
Right foot ulcer due to Shewanella algae after two weeks of antibiotic therapy with ciprofloxacin.

**Figure 3:**
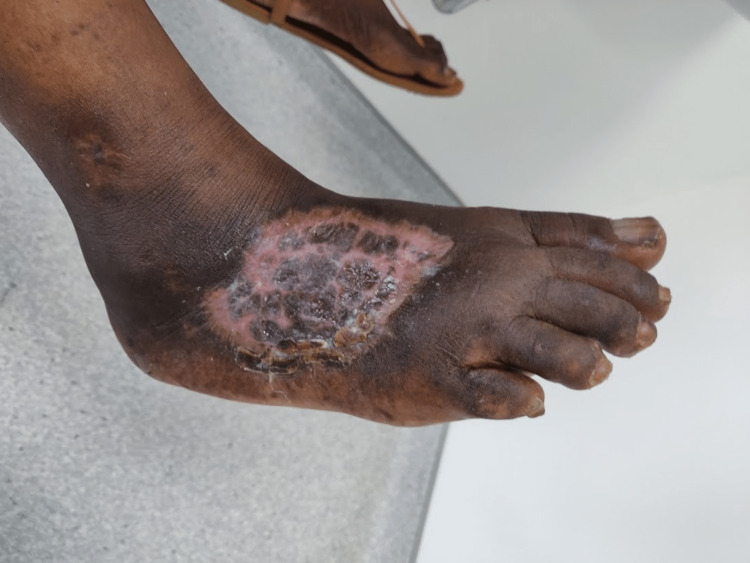
Right foot ulcer due to Shewanella algae after six weeks of antibiotic therapy with ciprofloxacin and a skin grafting.

A female patient is 56 years old and was born in São Tomé and Principe. She has a medical history of human immunodeficiency virus (HIV) infection, diagnosed seven years ago when she moved to Portugal. At the time of diagnosis, the patient’s CD4 T lymphocyte count was 34 cells/μL with no opportunistic infections. The patient was started on antiretroviral therapy (ART) with lamivudine/tenofovir disoproxil fumarate (FTC/TDF) plus dolutegravir (DTG). The patient stopped attending follow-up appointments, and a few months later she was admitted to the hospital’s emergency department, complaining of pain from an ulcer in her right foot that had begun three months prior (Figure 1). After a brief inquiry, she informed the medical team that she had been in São Tomé and Principe for the previous nine months and that she stopped ART during that period. In São Tomé and Principe, the patient lived at her family’s home and was in contact with seawater and river water every day for cleaning and housekeeping purposes. A blood test revealed a haemoglobin level of 11.0 g/dL, a white blood cell count of 8.2 × 103 cells/μL, and a C-reactive protein level of 3.0 mg/dL. The patient’s HIV-1 viral load was 79,300 copies/mL, and her CD4 T lymphocyte count was 240 cells/μL. The medical team collected pus from the ulcer for microbiologic analysis, and *S. algae* was isolated. The patient was treated with ciprofloxacin for 6 weeks, and ART was reinstated. The patient was evaluated by a plastic surgeon, and a skin graft was performed four weeks later. Figure 2 depicts the ulcer two weeks after the initiation of antibiotic treatment, and Figure 3 depicts the ulcer after the conclusion of the six-week antibiotic treatment and skin grafting. The patient currently maintains regular follow-up appointments at the infectious disease clinic.

Case 2

A female patient is 60 years old and was born in Guinea-Bissau, where she worked in rice fields and had daily contact with water. The patient had moved to Portugal two weeks prior to her admission to the emergency department, where she presented with a history of fever for the previous 15 days, weight loss, and fatigue. The patient’s medical history included chronic venous insufficiency, diagnosed five years prior. At the time she visited the emergency department, an ulcer with a diameter of 3 cm that had begun four months earlier, was visible on her left foot. A blood test revealed a haemoglobin level of 10.2 g/dL, a white blood cell count of 3.2 × 103 cells/μL (81% neutrophils), a platelet count of 190 × 103 cells/μL, and a C-reactive protein level of 12.0 mg/dL with normal liver and kidney function. The patient’s HIV antibody tests were also positive, and a diagnosis of HIV-1 was made. The HIV-1 viral load was 7180 copies/mL, and the CD4 T lymphocyte count was 167 cells/μL. *S. algae* was isolated from blood cultures and from a swab containing pus from the ulcer. The patient was medicated with cefotaxime and gentamicin for 10 days and started on ART with FTC/TDF plus DTG. At a follow-up appointment one month later, the patient no longer had a fever, inflammatory biomarkers were negative, and the ulcer was healing.

Case 3

A female patient is 74 years old and was born in Portugal, where she lived her whole life. She had no recent history of travel abroad. Medical history comprised stage 5 chronic kidney disease under dialysis, type 2 diabetes, arterial hypertension, and aortic stenosis. Additionally, the patient was on a follow-up study for chronic pancreatitis. She visited the emergency department complaining of a fever (39.5 °C) and confusion; her symptoms had begun less than 48 hours earlier. Initial blood tests revealed a haemoglobin level of 8.2 g/dL, a white blood cell count of 24.2 × 103 cells/μL, a platelet count of 52 × 103 cells/μL, a C-reactive protein level of 25.3 mg/dL, an aspartate transaminase level of 158 U/L, an alanine transaminase level of 226 U/L, a lactate level of 4.2 mmol/L, a D-dimer level of 3.5 µg/mL, and a fibrinogen level of 162 mg/dL. The patient quickly progressed to septic shock and was admitted to the intensive care unit (ICU), where mechanical ventilation and vasopressor therapy with noradrenaline were promptly initiated. Empirical antibiotic therapy was started with meropenem and vancomycin. *S. algae* was isolated 48 hours after hospital admission from two sets of blood cultures. No epidemiological risk factors were found. The patient died less than 48 hours postadmission to the ICU with disseminated intravascular coagulation.

Case 4

A male patient is 72 years old and was born in Portugal, where he lived his whole life. He had no recent history of travelling abroad. He had a medical history of hypertensive and alcoholic dilated cardiomyopathy, atrial fibrillation, chronic hepatic disease secondary to past alcohol consumption, obesity, and chronic venous insufficiency. He visited the emergency department with congestive heart failure with anasarca. Additionally, a 3.5-cm-diameter ulcer on the patient’s right foot with cellulitis in the surrounding tissues was visible. Initial blood tests revealed a haemoglobin level of 9.6 g/dL, a white blood cell count of 12.2 × 103 cells/μL, a platelet count of 156 × 103 cells/μL, a C-reactive protein level of 16.3 mg/dL, and an NT-proBNP level of 21,540 pg/mL. Pus was collected from the ulcer, and Klebsiella pneumoniae, Enterobacter cloacae, and *S. algae* were isolated. Blood cultures were negative, and no epidemiological risk factors were found. The patient started therapy with piperacillin and tazobactam. He died 72 hours after admission to the ward due to acute pulmonary oedema.

In all cases, light brown-raised colonies grew on the MacConkey agar medium, consistent with descriptions from the literature [2]. *S. algae* was identified by matrix-assisted laser desorption ionization-time of flight mass spectrometry. Although this technique has some limitations in identifying Shewanella at the species level due to an incomplete database [5-9], new efforts to create a new database have produced promising results [10]. Susceptibility testing was not performed on any of the isolates. As this is a retrospective study from the past 10 years, images for cases 2, 3, and 4 could not be obtained.

## Discussion

Looking at the most extensive study to date, *S. algae* was isolated from 118 patients with associated risk factors, including the following: hepatobiliary diseases, malignancy, chronic kidney disease, and diabetes mellitus [6]. Other risk factors described in the literature include male sex [11], immunodeficiency, and chronic ulcerations on the lower limbs [5-12]. The cases presented in this paper are consistent with the literature. Cases 1 and 2 deserve particular attention because they represent patients with HIV infection and low CD4 T lymphocyte cell counts arriving from African countries. Immigration from such countries is increasing, and HIV prevalence is higher amongst migrants [13], thus indicating a potential risk for an increased number of cases of *S. algae* infection.

Possible pathways for infection include recreational and occupational exposure, seafood ingestion, puncture wounds caused by marine organisms, and direct exposure of a wound to an aquatic environment [7]. Notably, a possible epidemiological linkage to infection was found in cases 1 and 2, but not in cases 3 and 4, highlighting that patients can be infected with *S. algae* even without contact with fresh water, seawater, or any other known risk factor.

Most clinically relevant infections include otitis media, skin and soft tissue infections (usually following local trauma or ulceration), invasive diseases (usually uncomplicated bacteraemia), and hepatobiliary diseases [5-7]. Other infections, though rarely described, have been identified, including some with a fulminant course [14]. In our case series, case 3 represents a patient with a fulminant course of infection. Although *S. algae* is able to survive in fomites and the patient was under haemodialysis, nosocomial infection is extremely rare. Most likely, the infection was acquired outside of healthcare settings. The patient’s comorbidities play a major role as risk factors for the fulminant course of the infection, but in the end, bacteremia leading to septic shock was the cause of death.

*S. algae* is generally susceptible to third- and fourth-generation cephalosporins, beta-lactam/beta-lactamase inhibitors, carbapenems, aminoglycosides, fluoroquinolones, and erythromycin [2,15], consistent with the results from recent papers [6-7]. Notably, antibiotic resistance to *S. algae* is increasing (namely among carbapenems, cephalosporins, and fluoroquinolones [6,7,11]), and multiple determinants of antibiotic resistance have been detected in the genes [15]. A recent whole-genome analysis of *S. algae* found eight classes of antibiotic-associated resistance genes, including β-lactam resistance genes, in 100 % of the isolates [16]. To date, the European Committee on Antimicrobial Susceptibility Testing has not defined clinical breakpoints for susceptibility testing, indicating the reason the microbiology laboratory did not perform susceptibility tests on *S. algae* isolates from the patients in this report. To date, these are the first cases of infection from *S. algae* reported in Portugal, including two cases wherein the infection was likely acquired in Portuguese territory, demonstrating not only that the infection exists in Portugal but also that it is likely underreported.

## Conclusions

*Shewanella algae* infection is an emerging human pathogen that has been increasingly reported. Clinicians should be aware of the clinical presentation and risk factors of this infection to effectively identify and treat it. The potential for antibiotic resistance is considerable, thus increasing the importance of treating the infection with proper antibiotic therapy. Increasing numbers of migrants from tropical areas may lead to an increasing number of *S. algae* isolates. The four clinical cases presented in this report highlight key clinical and epidemiological features of *S. algae*.
